# Multi-omics Data Integration, Interpretation, and Its Application

**DOI:** 10.1177/1177932219899051

**Published:** 2020-01-31

**Authors:** Indhupriya Subramanian, Srikant Verma, Shiva Kumar, Abhay Jere, Krishanpal Anamika

**Affiliations:** 1 LABS, Persistent Systems, Pune, India; 2 Innovation Cell, Ministry of Human Resource Development, New Delhi, India

**Keywords:** multi-omics, data integration, disease subtyping, biomarker prediction, data repositories

## Abstract

To study complex biological processes holistically, it is imperative to take an integrative approach that combines multi-omics data to highlight the interrelationships of the involved biomolecules and their functions. With the advent of high-throughput techniques and availability of multi-omics data generated from a large set of samples, several promising tools and methods have been developed for data integration and interpretation. In this review, we collected the tools and methods that adopt integrative approach to analyze multiple omics data and summarized their ability to address applications such as disease subtyping, biomarker prediction, and deriving insights into the data. We provide the methodology, use-cases, and limitations of these tools; brief account of multi-omics data repositories and visualization portals; and challenges associated with multi-omics data integration.

## Introduction

Comprehensive understanding of human health and diseases requires interpretation of molecular intricacy and variations at multiple levels such as genome, epigenome, transcriptome, proteome, and metabolome. With the advent of sequencing technology, biology has become increasingly dependent on data generated at these levels, which together is called as “multi-omics” data. Availability of multi-omics data has revolutionized the field of medicine and biology by creating avenues for integrated system-level approaches.

Analysis of multi-omics data along with clinical information has taken the front seat in deriving useful insights into the cellular functions. Integration of multi-omics data providing information on biomolecules from different layers seems to be promising to understand the complex biology systematically and holistically.^1^ Integrated approaches combine individual omics data, in a sequential or simultaneous manner, to understand the interplay of molecules.^2^ They help in assessing the flow of information from one omics level to the other and thus help in bridging the gap from genotype to phenotype. Integrative approaches, by virtue of their ability to study the biological phenomenon holistically, have the ability to improve prognostics and predictive accuracy of disease phenotypes and hence can eventually aid in better treatment and prevention.^1,3^

In recent times, various studies have shown that combining omics data sets yield better understanding and clearer picture of the system under study. For instance, integrative analysis of ChIP-Seq and RNA-Seq data of head and neck squamous cell carcinoma (HNSCC) cell lines showed that cancer-specific histone marks, H3K4me3 and H3K27ac, are associated with transcriptional changes in HNSCC driver genes, epidermal growth factor receptor (EGFR), FGFR1, and FOXA1.^4^ Zhang et al^5^ showed the importance of integrating proteomics data along with genomic and transcriptomic data to prioritize driver genes in colon and rectal cancers. Their results showed that chromosome 20q amplicon was associated with the largest global changes at both messenger RNA (mRNA) and protein levels. Integration of proteomics data helped in the identification of potential 20q candidates, including HNF4A (hepatocyte nuclear factor 4, alpha), TOMM34 (translocase of outer mitochondrial membrane 34), and SRC (SRC proto-oncogene, nonreceptor tyrosine kinase).^5^ In another study, integrating metabolomics and transcriptomics yielded molecular perturbations underlying prostate cancer. The metabolite sphingosine demonstrated high specificity and sensitivity for distinguishing prostate cancer from benign prostatic hyperplasia, as reported in this study. Downstream of sphingosine, the impaired sphingosine-1-phosphate receptor 2 signaling represents a loss of tumor suppressor gene and a potential key oncogenic pathway for therapeutic targeting.^6^

These studies widely proved the importance of integrating multi-omics data over single omics analysis. Employment of multi-omics approach has resulted in the development of various tools, methods, and platforms provisioning multi-omics data analysis, visualization, and interpretation. There are various review articles that cover the importance of multi-omics approaches from different perspectives. Multiple reviews are available that provide a summary of the multi-omics data integration methodologies categorized based on their underlying mathematical aspects.^2,7-9^ Yan et al^1^ summarize the network-based approaches used for multi-omics data analysis, whereas Tini et al^10^ provide benchmarking of unsupervised clustering methods in data integration.

In this review, we focus on the tools and methods that perform integration of multiple omics data and discuss in detail about their applications in understanding the complex human biology. The tools are chosen based on the below-mentioned criteria:

1. The approach must perform an integrative step wherein multiple data sets are analyzed in a simultaneous manner (parallel integration of data sets and not sequential). Platforms such as Galaxy^11^and O-Miner^12^ that help in analyzing multi-omics data, albeit individually, are not part of this review.
2. The approach must integrate at least 2 omics data sets derived from samples that have at least partial overlap.
3. The method or approach should be readily available in the form of tool/package to be able to execute the method on any data set.

In the following sections, the tools/methods are classified based on their ability to address diverse biological case studies showcased in their publications using multi-omics data. We also provide a detailed account of various portals that allow visualization of multi-omics data sets along with analysis that aids in understanding the correlation between the omics data sets.

## Omics Data Types and Repositories

Multi-omics data broadly cover the data generated from genome, proteome, transcriptome, metabolome, and epigenome. The spectrum of omics can be further extended to other biological data such as lipidome, phosphoproteome, and glycol-proteome. Multi-omics data generated for the same set of samples can provide useful insights into the flow of biological information at multiple levels and thus can help in unraveling the mechanisms underlying the biological condition of interest. There are a few publicly available databases, listed in Table 1, that provide multi-omics data sets of patients.

**Table 1.** List of multi-omics data repositories.

Data repository	Web link	Disease	Types of multi-omics data available
The Cancer Genome Atlas (TCGA)	https://cancergenome.nih.gov/	Cancer	RNA-Seq, DNA-Seq, miRNA-Seq, SNV, CNV, DNA methylation, and RPPA
Clinical Proteomic Tumor Analysis Consortium (CPTAC)	https://cptac-data-portal.georgetown.edu/cptacPublic/	Cancer	Proteomics data corresponding to TCGA cohorts
International Cancer Genomics Consortium (ICGC)	https://icgc.org/	Cancer	Whole genome sequencing, genomic variations data (somatic and germline mutation)
Cancer Cell Line Encyclopedia (CCLE)	https://portals.broadinstitute.org/ccle	Cancer cell line	Gene expression, copy number, and sequencing data; pharmacological profiles of 24 anticancer drugs
Molecular Taxonomy of Breast Cancer International Consortium (METABRIC)	http://molonc.bccrc.ca/aparicio-lab/research/metabric/	Breast cancer	Clinical traits, gene expression, SNP, and CNV
TARGET	https://ocg.cancer.gov/programs/target	Pediatric cancers	Gene expression, miRNA expression, copy number, and sequencing data
Omics Discovery Index	https://www.omicsdi.org	Consolidated data sets from 11 repositories in a uniform framework	Genomics, transcriptomics, proteomics, and metabolomics

Abbreviations: CNV, copy number variation; miRNA, microRNA; RPPA, reverse phase protein array; SNP, single-nucleotide polymorphism; SNV, single-nucleotide variant.

### The Cancer Genome Atlas

The Cancer Genome Atlas (TCGA; https://cancergenome.nih.gov/) houses one of the largest collections of multi-omics data sets for more than 33 different types of cancer for 20 000 individual tumor samples.^13^ This initiative aims to generate, merge, analyze, and interpret the profiles of DNA, RNA, protein, and epigenetic changes in tumor samples along with the clinical and histological data. It contains rich molecular and genetic profiles from primary tumor samples of various cancers and their subtypes. They generate high-throughput RNA-Seq, DNA-Seq, miRNA-Seq, single-nucleotide variant (SNV), copy number variation (CNV), DNA methylation, and reverse phase protein array (RPPA) data. Pan-cancer atlas is widely used by the research communities that have helped in making new discoveries about progression, manifestation, and treatment of cancers.^13,14^ The biospecimens from TCGA are analyzed by mass spectrometry technique, and the cancer cohort proteomics data are available at Clinical Proteomic Tumor Analysis Consortium (CPTAC) (https://cptac-data-portal.georgetown.edu/cptacPublic/).^15^

### International Cancer Genomics Consortium

International Cancer Genomics Consortium (ICGC; https://icgc.org/) coordinates large-scale generation of genome studies from 76 cancer projects in 21 primary cancer sites from 20 383 donors (as on December 2017). This project mainly contains mutation-related genomic alteration data (both germline and somatic) across cancer types from various ethnicity. The consortium defines the catalog for each tumor type and ensures quality of the data generated and manages data sharing across research communities. The ICGC Data Coordination Center (DCC) operates the ICGC data portal which contains both Open and Restricted access parts of the data.^16^ The ICGC portal has been used in deriving landmark observations in cancer biology.^17,18^ The Pan-cancer analysis of whole genomes (PCAWG; https://dcc.icgc.org/pcawg) allows the exploration and analysis of more than 2800 whole genomes from ICGC.

### Cancer Cell Line Encyclopedia

Cancer Cell Line Encyclopedia (CCLE; (https://portals.broadinstitute.org/ccle) hosted by Broad institute is a compilation of gene expression, copy number, and sequencing data from 947 human cell lines and for 36 tumor types. It also houses the pharmacological profiles of 24 anticancer drugs across 479 cancer cell lines. This project has enabled the identification of novel biomarkers and mechanistic effectors of drug response in different cancer cell lines.^19^

### Molecular Taxonomy of Breast Cancer International Consortium

Molecular Taxonomy of Breast Cancer International Consortium (METABRIC; http://molonc.bccrc.ca/aparicio-lab/research/metabric/) is a Canada-UK project that contains clinical traits, expression, single-nucleotide polymorphism (SNP), and CNV data derived from breast tumors. This project aims to classify breast tumors into further subcategories using the underlying multi-omics molecular signatures. This database identified 10 subgroups of breast cancer and new drug targets that were not previously described, and thus will help in designing the optimal course of treatment for breast cancer.^20^

### TARGET

TARGET (https://ocg.cancer.gov/programs/target), an initiative similar to TCGA, is driven by the National Cancer Institute (https://www.cancer.gov/) to determine the molecular events that drive childhood cancers.^21^ These data house the clinical information, gene expression, miRNA expression, copy number, and sequencing data of 24 molecular types of cancer. This database aims to provide a strong basis for functional assessment of genomic alterations across pediatric cancers.^21,22^

### Omics Discovery Index

Omics Discovery Index (OmicsDI; https://www.omicsdi.org/) contains data sets from 11 repositories in a common data structure. It is an open-source platform to access, discover, and integrate genomics, transcriptomics, proteomics, and metabolomics data sets. It contains data sets from humans, model organisms, and nonmodel organisms. Apart from indexing the data sets, OmicsDI also includes normalization and annotation step for every data set that can be integrated.^23^

Apart from these dedicated databases for multi-omics, National Center for Biotechnology Information (NCBI) Gene Expression Omnibus (GEO) archives a wide collection of sequencing data, such as genomics and transcriptomics, from multiple platforms and arrays.

## Leveraging Multi-omics Data to Derive Actionable Insights

Genes, transcripts, proteins, metabolites, and other macro/micro molecules systematically collaborate to perform complex cellular processes. It has been widely shown that integration of multi-omics data sets can help in unraveling the underlying mechanisms at multiple omics levels. Using TCGA data, previous reports identified distinct molecular subtypes of breast cancer by combining data from different layers such as CNV, mutation, DNA methylation, transcriptomics (mRNA expression and microRNA [miRNA] expression), and proteomics. The integrative analysis produced a comprehensive catalog of genetic and epigenetic drivers of breast cancer subtypes.^24^ Furthermore, Zheng et al showed that addition of proteomic data sets to genomic and transcriptomic data helped in deriving useful insights into high-grade serous ovarian cancer. This analysis showed that the integration of proteomics data complements genomics in the identification of multiple pathways and processes that drive ovarian cancer and potential drivers that can stratify patients for informed therapeutic management.^25^

Herein, we discuss in detail the tools and their methods that allow integration of multi-omics data sets to address the various challenges related to disease and their mechanisms. The tools are organized based on their ability to address biological question of interest. The biological questions are broadly categorized into 3 different case studies:

1. Disease subtyping and classification based on multi-omics profiles;
2. Prediction of biomarkers for various applications including diagnostics and driver genes for diseases;
3. Deriving insights into disease biology.

**Figure 1.**
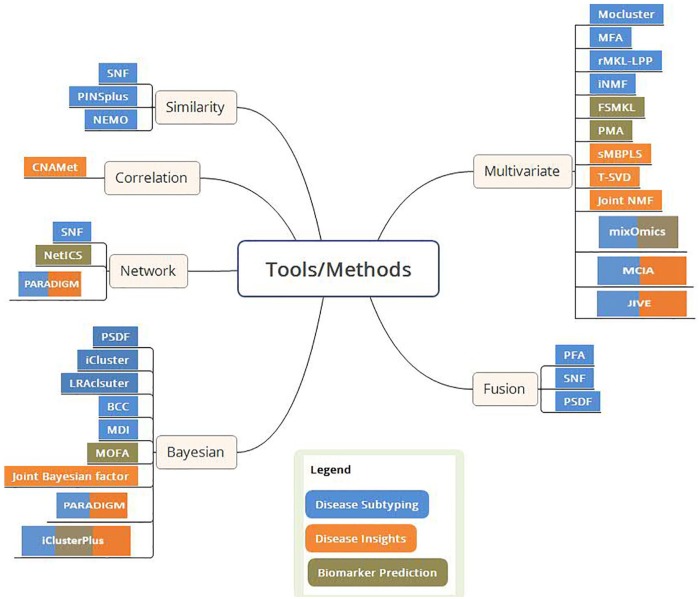
Overview of multi-omics data integration tools. The tools/methods are grouped based on their approach and are color coded as per their applications. FSMKL indicates feature selection multiple kernel learning; JIVE, joint and individual variation explained; MCIA, multiple co-inertia analysis; MDI, multiple dataset integration; MFA, multiple factor analysis; MOFA, multi-omics factor analysis; NEMO, neighborhood based multi-omics clustering; PFA, pattern fusion analysis; PMA, penalized multivariate analysis; sMBPLS, sparse multi-block partial least squares; SNF, similarity network fusion; NMF, nonnegative matrix factorization; BCC, Bayesian consensus clustering; PSDF, patient-specific data fusion.

The approach used by the tools or methods under each case study can be largely classified into one or more of the following categories: network, Bayesian, fusion, similarity-based, correlation-based, and other multivariate methods. Figure 1 provides a schematic representation of the integrative tools and methods grouped according to the approaches used. Few tools like PARADIGM, similarity network fusion (SNF), and so on use a combination of these approach categories as shown in Figure 1. The tools and methods under each case study are presented as per their approach categories. Tools falling under combination categories are explained only under the first appearing approach section. Table 2 summarizes the tools/methods, their approach, multi-omics data processed, and availability along with details of the input data used to showcase the applications of the tools. In this article, the data type numerical refers to continuous (for instance, segmentation mean data of comparative genomic hybridization [CGH] arrays) and discrete data (for instance, read counts in RNA-Seq) and categorical refers to all categorical data (for instance, ternary copy number data) including binary data. The presence of missing values in multi-omics data is inevitable and needs to be addressed by the data integration tools. Few of the tools mentioned in this article can handle missing data (refer Table 2) using imputation methods, whereas other tools require handling/removal of missing values in preprocessing steps.

**Table 2.** Integrative tools and methods addressing multi-omics applications, their usage and availability along with details of the input data used by each tool in their case study.

Use-case addressed	Tool/method	Tool/method approach	Supervised/unsupervised	Tool/method link	Tool/method language	Omics data and data type supported	Handling missing data by the tool	Disease studied in case study	Input data used in case study	No. of samples in input data	Input data source
Disease subtyping	PARADIGM	Probabilistic graphical models using directed factor graphs	Unsupervised	http://paradigm.five3genomics.com/	Python	Multi-omics (numerical)	NA	Glioblastoma multiforme	CNV segmentation; gene expression	230 patient samples and 10 adjacent normal tissues	McLendon et al., 2008^26^
Disease subtyping	iCluster	Joint latent variable model–based clustering method	Unsupervised	https://cran.r-project.org/web/packages/iCluster/	R package	Copy number; DNA methylation; gene expression; (numerical)	NA	Breast cancer	Copy number; gene expression	37 primary breast cancer and 4 breast cancer cell lines	Pollack et al^27^
Disease subtyping	iCluster	Joint latent variable model–based clustering method	Unsupervised	https://cran.r-project.org/web/packages/iCluster/	R package	Copy number; DNA methylation; gene expression; (numerical)	NA	Glioblastoma multiforme	Copy number; gene expression; DNA methylation	55 samples with all 3 data sets	McLendon et al., 2008^26^
Disease subtyping	iClusterPlus	Generalized linear regression for the formulation of a joint model	Unsupervised	https://bioconductor.org/packages/release/bioc/html/iClusterPlus.html	R package	Multi-omics (numerical and categorical)	NA	Colorectal cancer	Copy number; gene expression; DNA methylation; exome sequencing	189 colorectal carcinoma samples	TCGA
Disease subtyping	LRAcluster	Probabilistic model with low-rank approximation	Unsupervised	http://bioinfo.au.tsinghua.edu.cn/software/lracluster/	R package	Multi-omics (numerical and categorical)	NA	Cancer	Mutations; CNVs; DNA methylation; gene expression for 11 different cancers (BRCA, COAD, GBM, HNSC, KIRC, LGG, LUAD, LUSC, PRAD, STAD and THCA)	3319 samples	TCGA
Disease subtyping	PSDF	Data fusion by Bayesian nonparametric Dirichlet modeling	Unsupervised	https://sites.google.com/site/patientspecificdatafusion/	MATLAB	Copy number; gene expression (categorical)	NA	Breast cancer	Copy number; gene expression	106 samples	Chin et al^28^
Disease subtyping	PSDF	Data fusion by Bayesian nonparametric Dirichlet modeling	Unsupervised	https://sites.google.com/site/patientspecificdatafusion/	MATLAB	Copy number; gene expression (categorical)	NA	Prostate cancer	Copy number; gene expression	150 samples	Taylor et al^29^
Disease subtyping	BCC	Bayesian consensus clustering	Unsupervised	https://github.com/ttriche/bayesCC	R	Multi-omics (numerical)	No	Breast cancer	Gene expression; DNA methylation; miRNA expression and protein	348 breast cancer samples	TCGA
Disease subtyping	MDI	Bayesian models	Unsupervised	http://www2.warwick.ac.uk/fac/sci/systemsbiology/research/software/	MATLAB	Multi-omics (numerical and categorical)	NA	NA	NA	NA	NA
Disease subtyping	SNF	Local K-nearest neighbors (KNN); nonlinear method based on message-passing theory	Unsupervised	http://compbio.cs.toronto.edu/SNF/SNF/Software.html	R/MATLAB	Multi-omics (numerical and categorical)	No	Glioblastoma multiforme	mRNA expression; DNA methylation	215 patients’ GBM data	TCGA
Disease subtyping	PFA	Fusion method using PCA, k-means clustering	Unsupervised	http://www.sysbio.ac.cn/cb/chenlab/images/PFApackage_0.1.rar	MATLAB	DNA methylation; miRNA expression; gene expression; protein expression; (numerical)	No	Cancer	Gene expression; copy number	415 cell lines data	CCLE
Disease subtyping	PFA	Fusion method using PCA, k-means clustering	Unsupervised	http://www.sysbio.ac.cn/cb/chenlab/images/PFApackage_0.1.rar	MATLAB	DNA methylation; miRNA expression; gene expression; protein expression; (numerical)	No	Kidney renal clear cell carcinoma	Gene expression; miRNA expression; DNA methylation	122 KIRC samples	TCGA
Disease subtyping	PINSPlus	Similarity-based Clustering	Unsupervised	https://cran.r-project.org/web/packages/PINSPlus/index.html	R package	Multi-omics (numerical)	NA	Cancer	36 cancer data sets	3653 samples	TCGA and METABRIC
Disease subtyping	NEMO	Similarity-based Clustering	Unsupervised	https://github.com/Shamir-Lab/NEMO	R package	Multi-omics (numerical)	Yes	Acute myeloid leukemia	DNA methylation; miRNA expression; gene expression	Gene expression: 173 samples; miRNA expression: 188 samples; DNA methylation: 194 samples	TCGA
Disease subtyping; biomarker prediction	mixOmics	Supervised and unsupervised multivariate methods like PLS, SPLS, sGCCA, sPLSDA, and so on	Supervised & Unsupervised	http://mixomics.org/	R package	Multi-omics (numerical and categorical)	Yes	Breast Cancer	Gene expression; miRNA expression; Proteomics	150 samples	TCGA
Disease subtyping	moCluster	Consensus PCA (CPCA) approach	Unsupervised	https://bioconductor.org/packages/release/bioc/html/mogsa.html	R package	Multi-omics (numerical)	No	Colorectal cancer	Gene expression; DNA methylation; proteomics	83 colorectal cancer patients	TCGA; CPTAC
Disease subtyping	MCIA	Multiple co-inertia analysis	Unsupervised	http://bioconductor.org/packages/release/bioc/html/omicade4.html	R package	Multi-omics (numerical)	No	Ovarian cancer	Gene expression from multiple platforms (Agilent G4502A, Affymetrix HG-U133 2.0, Illumina HiSeq)	266 ovarian cancer gene expression data	TCGA
Disease subtyping	JIVE	Decomposition method with low-rank approximations	Unsupervised	https://genome.unc.edu/jive/	MATLAB	Multi-omics (numerical)	Yes	Breast cancer	DNA methylation; miRNA expression; gene expression	348 samples	TCGA
Disease subtyping	MFA	Multiple factor analysis	Unsupervised	http://factominer.free.fr/	R package	Multi-omics (numerical and categorical)	Yes	Glioma	CGH-array; gene expression	43 samples	Bredel et al^30^
Disease subtyping	rMKL-LPP	Multiple Kernel Learning	Unsupervised	Executable available on request	NA	Multi-omics (Numerical)	No	Glioblastoma multiforme	DNA methylation; miRNA expression; gene expression	213 samples	TCGA
Disease subtyping	iNMF	NMF	Unsupervised	https://github.com/yangzi4/iNMF	Python	Multi-omics (numerical)	No	Ovarian cancer	Gene expression, DNA methylation; miRNA expression	592 samples	TCGA
Disease subtyping; biomarker prediction; disease insights	iClusterPlus	Generalized linear regression for the formulation of a joint model;	Unsupervised	https://bioconductor.org/packages/release/bioc/html/iClusterPlus.html	R package	Multi-omics (numerical and categorical)	NA	Cancer	Copy number; gene expression; mutation	729 human cell lines representing 30 tumors	CCLE
Biomarker prediction	MOFA	Probabilistic Bayesian model	Unsupervised	https://github.com/bioFAM/MOFA	R/Python	Multi-omics (numerical and categorical)	Yes	Chronic lymphocytic leukemia	Mutation; gene expression, DNA methylation; drug response data (63 drugs)	200 samples of leukemia and lymphoma	Dietrich et al^31^
Biomarker prediction	NetICS	Network diffusion method	Unsupervised	https://github.com/cbg-ethz/netics	MATLAB	Multi-omics (numerical and categorical)	NA	Cancer	Somatic mutations; CNVs; gene expression; miRNA expression for 5 different cancers (uterine corpus endometrial carcinoma [UCEC], liver hepatocellular carcinoma	UCEC: 560 samples; LIHC: 377 samples; BLCA: 412 samples; BRCA: 1098 samples; LUSC: 504 samples	TCGA
Biomarker prediction	FSMKL	Kernel based machine learning	Supervised	https://github.com/jseoane/FSMKL	MATLAB	Multi-omics (numerical and categorical)	NA	Breast cancer	Copy number; gene expression	2000 samples	METABRIC
Biomarker prediction	PMA	Supervised and unsupervised methods such as sparse CCA, sparce mCCA and sparse sCCA; multivariate methods using CCA	Supervised & Unsupervised	https://cran.r-project.org/web/packages/PMA/index.html	R package	Multi-omics (numerical and categorical)	Yes	Diffuse large B-cell lymphoma	CGH-array; gene expression	203 samples	Lenz et al^32^
Disease insights	PARADIGM	Probabilistic graphical models using directed factor graphs	Unsupervised	http://paradigm.five3genomics.com/	Python	Multi-omics (numerical)	NA	Breast cancer	Gene expression; copy number data	171 patients data	Public data sets from GEO, ArrayExpress and published studies
Disease insights	Joint Bayesian factor	Joint Bayesian factor	Unsupervised	https://sites.google.com/site/jointgenomics/	MATLAB	Multi-omics (numerical)	No	Ovarian cancer	DNA methylation; copy number; gene expression	74 samples	TCGA
Disease insights	CNAmet	Correlation between copy number, methylation, and gene expression using permutation test	Unsupervised	http://csbi.ltdk.helsinki.fi/CNAmet/	R package	Copy number; DNA methylation; gene expression (numerical and categorical)	NA	Glioblastoma multiforme	Copy number; gene expression; DNA methylation	50 samples	TCGA
Disease insights	MCIA	Multiple co-inertia analysis	Unsupervised	http://bioconductor.org/packages/release/bioc/html/omicade4.html	R package	Multi-omics (numerical)	No	Cancer	Gene expression; protein expression of NCI-60 panel of leukemia, lymphomas, melanomas, and carcinomas from 9 different tissues	59 cancer cell lines data	CELLMINER (http://discover.nci.nih.gov/cellminer/home.do); http://wzw.tum.de/proteomics/NCI60/; https://www.proteomicsdb.org
Disease insights	JIVE	Decomposition method with low-rank approximations	Unsupervised	https://genome.unc.edu/jive/	MATLAB	Multi-omics (numerical)	Yes	Glioblastoma multiforme	miRNA expression; gene expression	234 samples	TCGA
Disease insights	sMBPLS	Sparse multi-block PLS	Supervised	http://zhoulab.usc.edu/sMBPLS/smbpls_dl.htm	MATLAB	Multi-omics (numerical)	NA	Ovarian cancer	CNV; DNA methylation; miRNA expression; gene expression	230 samples	TCGA
Disease insights	T-SVD	Single vector decomposition	Supervised	http://web.stanford.edu/~xm24/tsvd_website/	R package	Multi-omics (numerical)	NA	Ovarian cancer	miRNA expression; gene expression	487 samples	TCGA
Disease insights	Joint NMF	NMF	Semi-supervised	https://academic.oup.com/nar/article/40/19/9379/2414808	MATLAB	Multi-omics (numerical)	NA	Ovarian cancer	DNA methylation; miRNA expression; gene expression	385 samples	TCGA

Abbreviations: BCC, Bayesian consensus clustering; BLCA, bladder urothelial carcinoma; CCA, canonical correlation analysis; CCLE, Cancer Cell Line Encyclopedia; CGH, comparative genomic hybridization; CNV, copy number variation; CPTAC, Clinical Proteomic Tumor Analysis Consortium; FSMKL, feature selection multiple kernel learning; GEO, Gene Expression Omnibus; BRCA, Breast Invasive Carcinoma; COAD, Colon Adenocarcinoma; HNSC, head and neck squamous cell carcinoma; JIVE, joint and individual variation explained; KIRC, kidney renal clear cell carcinoma; LGG, low-grade glioma; LIHC, liver hepatocellular carcinoma LUAD, lung adenocarcinoma; mCCA, multiple canonical correlation analysis; LUSC, lung squamous cell carcinoma; PRAD, Prostate adenocarcinoma; STAD, Stomach adenocarcinoma; THCA, Thyroid cancer; MCIA, multiple co-inertia analysis; MDI, multiple dataset integration; MFA, multiple factor analysis; miRNA, microRNA; MOFA, multi-omics factor analysis; NEMO, neighborhood based multi-omics clustering; NMF, nonnegative matrix factorization; PCA, principal component analysis; PFA, pattern fusion analysis; PLS, partial least squares; PMA, penalized multivariate analysis; rMKL-LPP, Regularized multiple kernel learning- locality preserving projections; sCCA, supervised canonical correlation analysis; sMBPLS, sparse multi-block partial least squares; SNF, similarity network fusion; SPLS, sparse partial least squares; sGCCA, sparse Generalized Canonical Correlation Analysis; sPLSDA, sparse partial least squares discriminant analysis; TCGA, The Cancer Genome Atlas; T-SVD, thresholding singular value decomposition.

Numerical data type includes continuous (for instance, segmentation mean data of CGH–arrays) and discrete data (for instance, read counts in RNA–Seq), and categorical data type includes all categorical data (for instance, ternary copy number data) including binary data. Missing values are marked “Yes” if the tool handles missing data, “No” if the tool requires missing value handling in preprocessing steps, and “NA” when the information is not available.

### Disease subtyping and classification of samples based on their omics profiles

Many diseases, especially cancer, are heterogeneous because of the remarkable degree of differences between cancer progression in affected individuals. In addition to this, multiple other factors such as environment and life style may play a role in disease heterogeneity. Hence, it is imperative to identify the underlying subtypes of a disease or classifying samples into known subgroups to understand the etiology of the disease and identify suitable interventions for patients belonging to different subtypes.^33-35^ There exist several tools that leverage multi-omics data from samples to identify subtypes of a disease or classify various samples into subgroups based on their omics profiles. In this section, we discuss the tools that help toward understanding the subgrouping of samples based on the underlying molecular patterns.

#### Bayesian approach

##### Pathway Recognition Algorithm using Data Integration on Genomic Models (PARADIGM)

Pathway Recognition Algorithm using Data Integration on Genomic Models infers the activities of patient-specific biological pathways from multi-omics data.^36^ Multiple omics-scale measurements on a single patient sample are combined to infer the activities of genes, their products, and abstract biological processes derived from curated pathway interactions from NCI Protein Interaction Database. PARADIGM uses Bayesian factor graphs and hence will also fall in the “Network” category.

A gene is modeled by a factor graph as a set of interconnected variables encoding the expression and known activity of a gene and its products, allowing the incorporation of many types of omics data as evidence. PARADIGM produces a matrix of Integrated Pathway Activities (IPAs) *A*, where *A_ij_* represents the inferred activity of entity *i* in patient sample *j*.

The PARADIGM integrative approach using gene expression and copy number data from TCGA Glioblastoma (GBM) revealed 4 subtypes of the disease (Table 2).^36^ The fourth subtype showed an interesting distinct pattern with downregulation of HIF-1-alpha transcription factor network and overexpression of the E2F transcription factor network. The inactivity of the HIF-1-alpha might be a marker that the tumors were more oxygenated, suggesting that they might be smaller or newer tumors. Upregulation of E2F, which acts with the retinoblastoma tumor suppressor, was consistent with an active suppression of cell cycle progression in the tumor samples of this subtype. In addition, this subtype was significantly different from the other clusters for their survival. In contrast, 2 of the first 3 subtypes had elevated EGFR signatures and an inactive mitogen-activated protein kinase cascade involving the GATA interleukin transcriptional cascade.^36^ Thus, PARADIGM IPAs provide a meaningful set of profiles for delineating subtypes with markedly different survival outcomes.

##### iCluster

iCluster method aims to generate a single cluster assignment for samples based on simultaneous inference from multiple data types.^37^ This unsupervised method uses a joint latent variable model for integrative clustering and incorporates flexible modeling of the associations between different data types and the variance-covariance structure within data types in a single framework while simultaneously reducing the dimensionality of the data sets. Likelihood-based inference is obtained through the expectation-maximization algorithm.^37^

By integrating the copy number and gene expression data (Table 2), iCluster helped in identifying novel subgroups and their characteristic molecular patterns in breast cancer. In breast cancer analysis, 4 cell lines (BT474, T47D, MCF7, and SKBR3) were grouped in cluster 1, differentiating them from the tumor samples. HER2/ERBB2 subtypes were observed in cluster 2, whereas a novel subtype showing amplifications in the end of the q-arm of chromosome 17 was grouped in cluster 3. Cluster 4 did not show a significant distinct pattern.^37^ Similarly, using copy number, gene expression, and methylation data sets of GBM, clustering analysis resulted in 3 distinct subtypes. The subtype represented by cluster 1 showed an unevenly distributed profile of copy number alterations, hypermethylation of genes involved in brain development and neuronal differentiation, and a proneural expression profile. The subtype shown as cluster 2 was characterized by association with EGFR alteration, gains of chromosome 19 and 20, methylation of homeobox genes, and enriched expression. The subtype shown as cluster 3 was characterized by NF1 and PTEN alterations and exhibits mesenchymal-like expression.^38^

However, it is not equipped to handle both categorical and continuous variables that are addressed in its advanced version, iClusterPlus.^39^

##### iClusterPlus

iClusterPlus is an enhancement of iCluster and uses generalized linear regression for the formulation of a joint model of categorical and numerical (continuous and count) variables from integrated genomic, epigenomic, and transcriptomic profiling. This method uses a set of latent variables to represent “k” driving factors which predict the key genomic variables and thus capture the biological variation. Furthermore, using Lasso regression approach, iClusterPlus pinpoints the subset of features that contribute to the biological variation between the subtypes.^39^

Using mutation, copy number, and gene expression profiles of 729 cancer cell lines representing 23 tumor types from CCLE (Table 2),^19^ iClusterPlus identified 12 distinct clusters. Although many cell lines were majorly grouped by their cell-of-origin for few cancer types (eg, small-cell lung carcinoma [SCLC], hematopoietic and lymphoid tissue, and breast cancer), several other subgroups were also revealed that were not lineage-dependent and possibly were driven by a shared genetic alteration (eg, cluster 9 which belonged to both non–small-cell lung cancer [NSCLC] and pancreatic cancer cell lines showed prevalent KRAS mutations).^39^

In another case study using TCGA colorectal carcinoma (CRC) data set of mutation, DNA copy number, promoter methylation, and gene expression from 189 samples (Table 2), iClusterPlus helped in the discovery of 2 new subtypes in addition to the 2 classic subtypes (chromosomally stable or unstable) based on chromosomal instability (CIN). The new subtypes CIN-negative showed the lowest degree of alteration (3% genome altered) and CIN-low showed moderate degree of alteration (14% genome altered).^39^

However, a limitation of this method is that statistical inference (statistical selection of the final model) is not straightforward owing to its computationally intensive approach and the use of penalized regression.^39^

##### LRAcluster

LRAcluster uses a probabilistic model with low-rank approximation method to find the principal low-dimension subspace for classification of omics data.^40^ In this method, each omics data is conditional on a size-matched parameter matrix and this low-rank parameter matrix can be represented in a low-dimensional space. The user-defined dimension parameter *r* (based on explained variance of the data) and the number of clusters (based on silhouette values) help in faster dimension reduction and better clustering of disease subtypes.^40^

LRAcluster was used to classify TCGA data sets containing 11 different types of cancer using 4 different omics data, namely, mutation, CNV, DNA methylation, and gene expression (Table 2). LRAcluster analysis yielded 10 clusters in a 10-dimensional space with samples from the same type of cancer grouped together in individual clusters. The 2 different types of brain cancer (low-grade glioma [LGG] and GBM) were grouped together in a cluster (Cluster 3). The HNSCC samples were observed in 2 different clusters (clusters 1 and 10). Cluster 10 also contains samples from lung squamous cell carcinoma (LUSC), indicating that the squamous carcinoma of different tissue origins can have common underlying molecular mechanisms.^40^ Thus, LRAcluster is able to perform an unsupervised clustering of samples using multi-omics data in a faster and efficient manner.

##### Patient-specific data fusion (PSDF)

This method uses a Bayesian nonparametric model (Dirichlet process mixture models) to integrate CNVs and gene expression data to stratify samples into sub-groups.^41^ Each sample is assigned a binary state based on their concordance between the 2 data sets. Only samples that show concordance are fused together, whereas the other samples remain unfused, thus accounting for patient-specific fusion models. Patient-specific data fusion (PSDF) uses Markov chain Monte Carlo (MCMC) sampling method to predict the probability for each sample that it is fused. The feature selection of PSDF helps in reducing the noise from data sets by selecting only those features that help in clustering. Feature selection is again a binary indicator and is identified for each data set separately. Patient-specific data fusion, thus, accounts for patient-specific consistent fusion and derives the number of clusters inherently.^41^ Although PSDF is explained under “Bayesian” section, it also uses “Fusion”-based approach.

Copy number and gene expression data of 106 breast cancer samples^28^ were clustered using the PSDF method (Table 2). One hundred six samples were grouped into 4 clusters, and 3 fused clusters were identified. Cluster 2 shows a distinct substructure where the expressions are distinctly different and the copy number is largely neutral. Few samples from clusters 1, 2, and 4 and all samples from cluster 3 have unfused samples. These samples have similar CNV pattern with a range of gene expression values. The survival analysis revealed a low survival group (cluster 1), a good outcome group (cluster 4), and intermediate groups (clusters 2 and 3). The features elected by PSDF show various well-reported genes in breast cancer. For instance, copy number features identified that 8q contains MYC, 17q contains BRCA1, and 17p encodes TP53.^41^ Similarly, the PSDF model identified 7 subtypes in prostate cancer data by integrating copy number and gene expression data from 150 tumor samples.^29,41^

##### Bayesian consensus clustering (BCC)

Consensus clustering is widely used to combine multiple clustering algorithms or to integrate multisource data sets. Bayesian consensus clustering (BCC) proposes a data-driven consensus clustering (CC) method that models source-specific features as well as an overall clustering using finite Dirichlet mixture model extended to account for multiple data sources.^42^ It forms separate clustering of individual data, but they are loosely connected to the overall clustering of all data sources. Bayesian consensus clustering performs both specific clustering and CC simultaneously, and CC is derived based on the distribution that gives higher probability to clusters that are present in specific regions. The authors also propose a heuristic approach to select the optimal number of clusters for a given data set. Bayesian consensus clustering implementation is based on the assumption that the data are normally distributed.^42^

The BCC method was applied to identify the subtypes of breast cancer using TCGA breast cancer data of 348 samples with gene expression, DNA methylation, miRNA expression, and protein data (Table 2). The method yielded 3 clusters that define the known subtypes of breast cancer. Cluster 1 of BCC corresponds to basal subtype, cluster 2 corresponds to Luminal A, and cluster 3 contained samples belonging to ER/PR-positive status. The specific patterns of gene expression data showed the highest adherence to overall clustering.^42^

##### Multiple dataset integration (MDI)

The multiple dataset integration method uses Dirichlet mixture models to cluster each data source while simultaneously modeling the pairwise dependencies between the clusters.^43^ The MDI links the models at the level of variables that are allocated to components such as genomic features. The component variable level linkage allows capture of dependencies between the multi-omics data. For instance, the method identifies a group of genes that are allocated to the same component that are clustered together across multiple data sets.^43^

Application of MDI as described by the authors is in *Saccharomyces cerevisiae* gene expression and ChIP data to identify the protein complexes whose genes are transcriptionally co-regulated.^43^ However, Savage et al^44^ and Chauvel et al^45^ have showcased the application of this method in the identification of disease subtypes using multi-omics data sets from TCGA.

#### Network approach

##### Similarity network fusion (SNF)

Similarity network fusion is a network-based approach to integrate multi-omics data sets using a network fusion method.^46^ First, SNF creates an individual network for each data type and then fuses these into a single similarity network using a nonlinear network fusion approach. The fusion step is based on message-passing theory that makes the network more like the others with each iteration. The advantage of this method is that the weak connections (noise) disappear with iterations, whereas the strong connections are propagated till convergence.^46^ Although this method is explained under “Network” approaches, SNF also uses “Fusion”- and “Similarity”-based techniques in its approach.

DNA methylation, miRNA expression, and gene expression of 215 GBM data samples from TCGA (Table 2) were integrated using SNF to identify the subtypes of GBM. The fused network identified 3 clusters that defined the previously reported subtypes of GBM. The smallest cluster (cluster 3) corresponds to reported IDH1 subtype containing younger patients with favorable prognosis. Cluster 1 corresponds to patients who responded to the GBM drug temozolomide (TMZ). Cluster 2 showed significant association with Cathepsin D (CTSD) overexpression, which is reported to prevent the effect of TMZ.^46^ Thus, SNF helps in identifying the subtypes of diseases using a novel network fusion approach.

#### Fusion-based approaches

##### Pattern fusion analysis (PFA)

Pattern fusion analysis (PFA) allows the identification of integrated sample patterns across heterogeneous genomic profiles in a low-dimensional feature space.^47^ Pattern fusion analysis obtains the local sample patterns using principal component analysis (PCA). Then, it aligns those local sample patterns to a common feature space and synthesizes the global sample pattern across most data types. During this process, the contributions by each data type (or individual sample) on the global sample spectrum would be quantitatively measured and the effects of bias or systematic noises would be iteratively decreased to better fit the data. The repeated correction will end when it reaches convergence. After the adaptive optimal alignment, the combinatorial sample pattern could represent comprehensive characterization, which would be closer to inherent relations in data. Thus, PFA helps in identifying distinct subgroups of cells or samples across multi-omics data set.

Analysis of the matched gene expression and copy number data of 415 cell lines representing 11 different tumor types from CCLE (Table 2) (Barretina, Caponigro and Stransky, 2012) obtained a 9-cluster sample pattern as an optimal solution. Upon analysis of the clusters for tumor-cluster enrichment ratios, they show that acute myelocytic leukemia (AML) and multiple myeloma have relatively high proportions of tissue/tumor-specific patterns and were clustered separately, whereas pancreatic LUSC and LUAD (lung adenocarcinoma) cell lines show great tumor heterogeneity. This is in concordance with the previous study by Mo et al.^39^

The PFA also identified 2 subtypes for kidney renal clear cell carcinoma (KIRC), 3 for LUSC and 3 subtypes for glioblastoma multiforme (GBM) based on gene expression, miRNA expression, and DNA methylation profiles from TCGA (Table 2). The 2 subtypes of KIRC showed significant difference in survival times, thus signifying the biological relevance of this method. The approach also identified previously reported important features of KIRC like CD44, ANXA2, and hsa-miR-21 overexpression associated with shorter survival times.^47^ Thus, these studies validate the potential of PFA to identify subgroups and aids in revealing functional associations based on multi-omics data.

However, this method does not support categorical data types (mutation/SNP) to be integrated and can make a weak fusion if most of the input data have consistent bias.^47^

#### Similarity-based approaches

##### PINSPlus

Perturbation clustering for data integration and disease subtyping (PINSPlus) is an unsupervised clustering method that helps in identifying subtypes from multi-omics data. To identify subtypes, the algorithm identifies how often the patients are grouped together in a single cluster (1) when the data are perturbed, (2) when using different types of omics data, and (3) when a different clustering technique is used. Strongly connected patients in all the scenarios are clustered together into a subtype.^48^

Patient connectivity for each data is represented in the form of graph with patients as node and connectivity as edges. Similarity matrix is generated by merging the connectivity from all data types, and similarity-based algorithm is used to identify subtypes. To identify subgroups within subtypes and to address the heterogeneous subgroup of patients within a subtype, a hierarchical structure search is performed. Thus, PINSPlus helps in the discovery of subgroups in a method and data-independent manner. The tool also allows customization of clustering methods based on user’s choice. PINSPlus is also reported to be fast and powerful to run on large omics data sets.^48^

PINSPlus was applied on 34 omics data sets from TCGA and 2 breast cancer data sets from METABRIC to identify the subtypes of cancers with differences in survival (Table 2). PINSPlus-identified subtypes for 27 of the 36 data sets showed significant *P* values between the subtypes for survival differences. For the remaining 9 cancer types, PINSPlus was not able to identify subtypes with different survival profiles.^48^

##### Neighborhood-based multi-omics clustering (NEMO)

Neighborhood-based multi-omics clustering (NEMO)^49^ is a similarity-based simple multi-omics clustering approach that further builds on previously established clustering methods such as SNF^46^ and rMKL-LPP.^50^ Neighborhood-based multi-omics clustering initially builds an interpatient similarity matrix–based Euclidean distance for each of the input omic data sets. The similarity matrix from each omics is then integrated into a single matrix, which is then clustered using the spectral clustering method. This method computes the multi-omics data integration and clustering in a simple and efficient manner compared with its counterparts. The major advantage of NEMO is that it is applicable on partial data sets, that is, some samples are measured only on subset of omics data.^49^

Neighborhood-based multi-omics clustering was applied on a partial AML data set from TCGA containing gene expression data from 173 samples, DNA methylation data from 194 samples, and miRNA expression data from 188 samples (Table 2). Five clusters that showed significant clinical outcomes were suggested, and the clusters were highly associated with the FAB (French-American-British) classification of AML samples. Cluster 1 showed favorable prognosis and contained young patients. Cluster 2 contained older patients with poor prognosis and majorly belonging to FAB level “M0 undifferentiated.” Cluster 3 showed favorable prognosis and enriched for FAB M3 label which corresponds to acute promyelocytic leukemia (APL). FAB M5 label samples were observed in cluster 4, and cluster 5 showed samples with no genetic aberrations. Thus, NEMO can cluster partial data sets to derive meaningful subtypes.^49^

#### Other multivariate approaches

##### mixOmics

mixOmics provides a set of supervised and unsupervised multivariate methods to perform integration of multi-omics data sets with focus on variable selection. This package allows integration of multi-omics data sets to classify or cluster samples using different methods such as PCA, independent principal component analysis (IPCA), partial least squares (PLS) regression, sparse partial least squares (SPLS) regression, canonical correlation analysis (CCA), and supervised analyses such as partial least squares discriminant analysis (PLS-DA). One of their frameworks, DIABLO, uses sPLSDA (sparse PLS-DA) method to identify highly correlated multi-omics signature to discriminate the subtypes of the disease.

Using expression data of gene, miRNA, and protein from 150 breast cancer samples available at TCGA (Table 2),^24^ DIABLO was showcased to identify multi-omics signatures (putative biomarkers) that could distinguish the breast cancer subtypes, namely, Basal, HER2, and Luminal A.^51^ Thus, mixOmics can address both disease subtyping and biomarker prediction.

##### moCluster

moCluster uses multi-table multivariate analysis approach to identify the patterns across multi-omics data sets.^52^ The first step of this approach involves identification of latent variables using sparse consensus PCA. The number of latent variables to be used in this model is determined using permutation and elbow test. Furthermore, the latent variables are clustered using traditional methods such as hierarchical or K-means and the selection of the best subtype model.

Analysis of DNA methylation, gene expression, and protein expression data from 83 samples of colorectal cancer from TCGA and CPTAC (Table 2) by moCluster resulted in 4 integrative subtypes, C1-C4. C1 represented a subtype which was associated with immune-related genes and proteins, and thus proposed to be well susceptible to drugs targeting immune checkpoint genes. Subtypes C2-C4 were not discovered in previous studies. C2 subtype was observed to have elevated ribosome biogenesis activity, and thus proposed to be associated with an increased risk of neoplastic transformation. C3 subtype was proposed to have a more epithelial phenotype and less metastatic potential compared with the C2 subtype. This clustering analysis showed that the CIN subtype of CRC can be further subdivided into 2 groups as these samples were observed to be part of both C2 and C4 integrated clusters. This provides a new basis to study the driving mechanisms and genes in colorectal cancer.^52^

##### Multiple co-inertia analysis (MCIA)

Multiple co-inertia analysis (MCIA) is an exploratory data analysis method that captures the co-relationships among multiple high-dimensional data sets (such as gene expression, miRNA expression, protein expression). The molecular features need not be present across all data sets; however, all data sets should have the same set of samples.

This approach uses a covariance optimization criterion to transform diverse sets of features (such as genes, proteins, miRNAs) onto the same scale and simultaneously projects multiple data sets into the same dimensional space. With the help of simple graphical representations, sample space, and feature space, one can efficiently identify the concordance between data sets and can extract features that are relevant to a sample cluster (representing a biological condition), respectively.^53^

Analysis of TCGA ovarian cancer gene expression data generated on microarray platforms and RNA-Seq platform for 266 samples (Table 2) yielded 4 subtypes that is, proliferative, immunoreactive, mesenchymal, and differentiated. This was achieved by visually observing the sample space in the first 2 MCIA axes, with the first axis separating samples with immunoreactive versus proliferative characteristics, and the second axis separating samples with a mesenchymal subtype from the differentiated subtype. Furthermore, examination of gene expression variables superimposed onto the same space could help to identify features specific to the 4 subtypes and hence is responsible for sample segregation. Thus, this case study highlights the potential of MCIA to identify disease subtypes.^53^

##### Joint and individual variation explained (JIVE)

This approach integrates multi-omics data by separating the joint and individual effects of the data sets. It uses a decomposition method and segregates the data sets into 3 terms, a low-rank approximation for the joint variation between data sets, a low-rank approximation for individual variations, and the residual noise.^54,55^ Joint and individual structure corresponds to *r* and *r_i_* dimensional subspace that explains variation across multiple data sets and within data sets, respectively. Permutation test is used to specify the ranks that help in quantifying the joint and individual patterns.^54,55^

The joint and individual variation explained (JIVE) method was applied on gene expression, DNA methylation, and miRNA data of 348 breast cancer samples from TCGA (Table 2). The point cloud view of samples in the reduced low-dimensional joint structure showed 3 clusters corresponding to the 3 subtypes of breast cancer. Cluster 1 separates basal-like breast cancer data from other samples. Cluster 2 corresponds to a subgroup of Luminal A with low fraction of genomics alteration and improved clinical prognosis.^55^

##### Multiple factor analysis (MFA)

Multiple factor analysis (MFA) is another method that helps in the integration of omics data sets by projecting it in a low-dimensional variable space.^56^ Multiple factor analysis allows integration of numerical variables and categorical variables that helps in the addition of supplementary group of data in the analysis. Multiple factor analysis provides a balanced representation of individual as well as common structures while data set integration. Principal component analysis is applied on each omic data to identify the individual pattern. Global analysis to identify the common structure involves identification of the variance-covariance matrix for each data set. It also provides the matrix of variables that allows visualization of individual and common structures. This method is implemented as one of the multivariate methods of FactomineR package in R.^56^

Multiple factor analysis was applied on CGH-array and transcriptome data sets for 43 glioma samples from GEO containing 4 types of glial tumors, namely, oligodendrogliomas, astrocytomas, mixed oligoastrocytomas, and glioblastomas (Table 2). PC1 summarizes the characteristics of glioblastoma samples from low-grade gliomas, whereas PC2 mainly differentiates oligodendrogliomas and astrocytomas. Analysis of genes involved in PC2 underlines genomic status alterations of genes on chromosome 1p and 19q, which are frequently reported in oligodendrogliomas. The authors also showcase the ability to integrate Gene Ontology Biological Process terms as supplementary data on the same principal components. With this, they identified the important biological process aligned with the PCs.^56^

##### rMKL-LPP

Regularized multiple kernel learning (rMKL) for dimensionality reduction uses multiple kernel learning for integration of heterogeneous multiple data and to perform subtype identification. The samples are projected in a low-dimensional space that can be used for clustering the samples. The method automatically assigns higher weights to high information content and avoids overfitting of model using a regularization term. Each input type is represented as a kernel matrix and also allows more than 1 kernel matrix for a data type to capture the different degrees of similarity within the data. Dimensionality reduction is achieved using locality preserving projections (LPP), an unsupervised method that clusters samples to its k-nearest neighbors.^50^

This method was applied for DNA methylation, miRNA expression, and gene expression of 5 different types of cancer (GBM, BRCA (Breast invasive carcinoma), Kidney renal clear cell carcinoma (KRCCC), COAD (Colon Adenocarcinoma), and Lung squamous cell carcinoma (LSCC)) from TCGA (Table 2). The clustering of samples for all cancers showed differences in survival and yielded better results than iCluster and SNF. The authors further compare the GBM clusters (using gene expression and DNA methylation of 213 samples) with existing subtypes derived only through gene expression data. Cluster 1 is enriched for mesenchymal, cluster 2 is mostly classical and neural subtype, and clusters 1 and 2 show weaker survival when treated with temozolomide. Proneural subtype is observed in clusters 3 and 4 wherein the G-CIMP status was positive in cluster 3 samples and negative in cluster 4. Samples in cluster 5 had increased survival time when treated with temozolomide.^50^

##### Integrative nonnegative matrix factorization (iNMF)

The nonnegative matrix factorization (NMF) method is widely used in analyzing high-dimensional data sets, and various extensions of this method are developed for better interpretation of multi-omics data. Integrative NMF extends the NMF framework to account for heterogeneous effects while integrating multiple data.^57^ Another extension of NMF, joint NMF (jNMF),^58^ allows identification of homogeneity in data sets while integration. Integrative NMF combines the homogeneous and heterogeneous pattern using a partitioned factorization structure which is a combination of NMF and jNMF objective functions. A novel tuning method of homogeneity parameter, λ, helps in accounting for heterogeneity in the data sets. As the objective function of iNMF is nonconvex, the method should be repeated many times to obtain the optimal minimal objective function.^57^

Integrative NMF was used on TCGA ovarian cancer data of 592 samples containing gene expression, DNA methylation, and miRNA expression (Table 2). The 4 clusters identified by iNMF (with λ = 0.01) correlated well with previously reported subtypes of ovarian cancer, immunoreactive (I), proliferative (P), differentiated (D), and mesenchymal (M).^59^ The modules pertaining to I and M showed discrepancies to previously established clusters, suggesting occurrence of heterogeneous noise patterns in these modules. This is captured as iNMF accounts for the heterogeneous noise patterns between data sets. Module I genes were mostly related to DNA repair and cell cycle regulation pathways; module P genes were related to proliferation and survival pathways; module D genes were associated with checkpoint regulation, survival, and cell migration; and module M genes were associated with the regulation of cell migration and tumor suppression, suggesting late stages of tumor development.^57^ These multidimensional modules (md-modules) that are correlated with published studies show the ability of iNMF to identify the molecular patterns underlying disease subtypes.

Apart from the above-mentioned tools and methods, the R package CancerSubtypes, provides a uniform framework to cluster multi-omics data sets to derive subtypes using 5 available methods and one of their in-house methods, namely, CC, consensus nonnegative matrix factorization (CNMF), iCluste, SNF, and weighted SNF (WSNF), along with a new combined method called SNF-CC. The first 2 methods are applicable for single data, whereas others can be used for multi-omics. The suite also allows validation analysis such as survival analysis, differential expression tests, silhouette width, and statistical significance of clustering to further validate and visualize the results.^60^

### Prediction of biomarkers for various applications including diagnostics and driver genes for diseases

Biomarkers are molecular footprints of the function of the cell in a condition of a living system. These biomolecules belong to strongly connected biological pathways that provide the flow of information, and thus can reveal the underlying biology. Integrative analysis offers a huge opportunity to identify reliable biomarkers based on data from multiple molecular events. As validation of biomarkers is time-consuming, an informed *in silico* approach–based nomination of biomolecules would be effective. In this section, we present the tools that allow interpretation of molecular features by combining multi-omics data sets that can drive the underlying biology of a disease. Widely, the methods use one of the feature selection methods to identify the distinct molecular pattern in a subtype or category.

#### Bayesian approach

##### iClusterPlus

In addition to disease subtyping, iClusterPlus also helps in identifying features associated with a subtype. iClusterPlus uses penalized likelihood approach with lasso penalty to associate a genomic feature with a phenotype. A genomic feature is associated with a subtype if the corresponding coefficient estimate is nonzero. As a result, clustering variability can be substantially reduced by effectively removing noninformative features by forcing their coefficients to zero.^39^

Using the CCLE data (Table 2), a gene-centric integration in each cluster accurately identified known drivers in several cancer types, including MITF in melanoma, ERBB2 in breast cancer, EGFR and MET in LUAD, and MYCN in brain tumors. These findings also highlight many candidate biomarkers or driver genes, including XPC, BAP1, and Scotin in small-cell lung cancer, and MYB and PCM1 in leukemia.^39^

Further improvement of iClusterPlus method with fully Bayesian model and improved computation time, iClusterBayes,^61^ has advanced the feature selection criterion and can help in the identification of prominent features from multi-omics data integration.

##### Multi-omics factor analysis (MOFA)

Multi-omics factor analysis (MOFA) is an unsupervised method for integrating multi-omics data types on the same or partially overlapped samples. This method helps in inferring an interpretable low-dimensional data representation as hidden factors on multiple modalities of omics data. It uses a probabilistic Bayesian framework for model formulation that can support combination of different noise models to integrate multiple data types such as numerical (continuous and count) and categorical (binary) data.^62^

A cohort of 200 samples with chronic lymphocytic leukemia (CLL) profiled for mutations, DNA methylation, gene expression, and drug response data (63 drugs) (Table 2) were used to validate MOFA’s ability in the identification of known and novel clinical markers.

Multi-omics factor analysis identified 10 hidden factors that captured major sources of variation across the multiple omics data, and thus helped in identification of continuous molecular gradients or discrete subgroups of samples. The first 2 major factors, factor 1 and factor 2, aligned with the 2 well-known and important clinical markers of CLL, IgHV mutation status and trisomy of chromosome 12, respectively, based on their loading weights in mutation data. Similarly, factor 5 aligned with a gene set (which includes heat-shock proteins) enriched for oxidative stress and senescence pathway, based on their loading weights in mRNA data. Drugs aligned with factor 5 were also shown to be associated with oxidative stress. This is an interesting observation as heat-shock proteins were not well known in the context of CLL.

It is important to note that the use of linear models in MOFA to represent relationships between data can fail to capture the strong nonlinear relationships between and within omics.^62^

#### Network approach

##### Network-based integration of multi-omics data (NetICS)

The network-based integration of multi-omics data (NetICS) method provides a framework for network-based integration of multi-omics data for cancer gene prioritization. It predicts the effect of genetic aberrations, epigenetic changes, and miRNAs on downstream genes and protein (expression) in the interaction network. It uses a per-sample network-diffusion model on a directed functional interaction network and derives a population-level gene ranking by aggregating individual rankings and provides a global ranking for all samples.^63^

Somatic mutations, CNVs, miRNA expression, and gene expression for 5 different cancers (uterine corpus endometrial carcinoma, liver hepatocellular carcinoma, bladder urothelial carcinoma, breast invasive carcinoma, and lung squamous cell carcinoma) from TCGA (Table 2) were analyzed in NetICS. This method ably identified both frequently and infrequently aberrant genes in the top-ranking genes. TP53 (frequent aberration), EP300, and AKT1 (infrequent aberration) were identified as top ranked in breast cancer data. Similarly, NetICS identified AKT1, EGFR, KRAS, NRAS, and PIK3CA among the top 5% in lung cancer data sets.^63^

However, NetICS can only analyze and examine the effect of genes that are present in the interaction network. Moreover, there is a possibility for bias toward highly connected genes in the network.^63^

#### Other multivariate approaches

##### Feature selection multiple kernel learning (FSMKL)

This supervised classification method uses multiple kernels to capture the similarity between data sets to identify features for disease progression. Each data set is encoded into a base kernel, a linear combination of which is used to create composite kernels. A large number of kernels are used with variable number of features per kernel per data type. Feature selection is achieved using statistical methods, and the algorithm finds the most relevant kernel and the features associated for a given classification problem. The kernel coefficients denote the significance of the kernel and thus are a measure of the importance/weight of the multiple data sets in the final decision function. This method allows incorporation of prior knowledge in the form of pathways such as KEGG in computing base kernels.^64^

This method was applied on METABRIC^20^ breast cancer expression and CNV data (Table 2) to predict the mortality risk and features associated with it. The following treatment groups were included—lymph node–negative without chemotherapy, ER-positive (hormone therapy), ER-negative (chemotherapy), and others. Apart from the genomic and transcriptomic data, adding the clinical variables associated with survival and ER status resulted in better predictions.^64^

##### Penalized multivariate analysis (PMA)

This R package consists of various versions of CCA that help in integrative analysis of multiple data sets measured from the same set of samples.^65^ The sparse CCA, sparse multiple CCA (sparse mCCA), and sparse supervised CCA (sparse sCCA) are the extensions of the CCA available in this package. The methods are aimed at extending CCA to include sparsity constraint (sparse CCA), outcome measurements when available (sparse sCCA), and more than 2 data sets while building the correlation between the data sets (sparse mCCA).

All the extensions of CCA were applied to gene expression and CGH-array measurements of 203 samples (Table 2) with diffuse large B-cell lymphoma (DLBCL).^32^ Sparse CCA was performed on the whole gene expression data and CGH data for a given chromosome “i.” The canonical variables obtained were highly correlated validating the sparsity constraint. Furthermore, the variables/features were highly predictive of the subtypes of DLBCL (germinal center B-cell like, activated B-cell like, and primary mediastinal B-cell lymphoma). Sparse mCCA approach was applied to analyze the effect of CGH measurements on the copy number changes in genomic regions. This analysis reveals that complex pattern of gain and loss tends to co-occur.^65^

Using the survival and subtype information of the 203 samples, sparse sCCA was performed on the data to predict the associated canonical variables. The variables from sparse sCCA had lower *P* values than those obtained from sparse CCA. However, the variables from both the methods were not significantly associated with survival.^65^

### Deriving insights into disease biology

Understanding the mechanistic details of disease biology lies central to diagnosis and developing novel interventions for the disease. In this section, we present the tools that leverage multi-omics data to derive insights into disease biology. We elaborate on the approach used by each tool and the ways (i.e, use-cases) in which these tools are used to derive insights.

#### Bayesian approach

##### PARADIGM

The application of PARADIGM method can be extended to derive findings into the disease under study. Wirapati et al^66^ showed the application of PARADIGM to derive novel insights into breast cancer using copy number and gene expression data (Table 2). In this analysis, 56 172 IPAs (7% of the total) were found to be significantly higher or lower than the matched negative control. On an average, 103 out of 127 NCI pathways had at least 1 entity altered in 20% or more of the patients. PARADIGM was able to detect the estrogen- and ErbB2-related pathways,^36^ which were found to be 2 of the 3 key prognostic signatures in breast cancer in a recent major meta-analysis study.^66^ It is important to note that PARADIGM also identified an AKT1-related PI3K signaling pathway as the top-most pathway with significant IPAs in several samples. The antiapoptotic AKT1 serine-threonine kinase is known to be involved in breast cancer and interacts with the ERBB2 pathway.^36^ Thus, the analysis helped in gaining additional insights into the biology of breast cancer.

##### iClusterPlus

Like PARADIGM, iClusterPlus also aids in deriving insights into diseases. In the previous section, we described the ability of this method to cluster cancer cell line data (Table 2). Furthermore, associating the integrated clusters with the pharmacological profiles of 24 anticancer drug compounds revealed selective sensitivity to MEK inhibitors in a subset of hematopoietic cell lines, a potentially clinically important finding that a subgroup of hematological malignancies may benefit from MEK inhibitors.^39^

##### Joint Bayesian factor

This method uses nonparametric Bayesian factor analysis to integrate omics data sets. This approach factorizes the feature space into shared and data-specific component using a beta-Bernoulli process.^67^ The joint factor model consists of the individual factor loadings specific to a data set and common factor loadings across all data sets and noise/residual specific to the data set. Student-*t* sparseness-promoting prior is used to add the sparsity to the factor loadings. This method allows the flexibility of discovering factors specific to a subset of samples that adds value to the proposed model.^67^

Joint Bayesian factor was applied to integrate the gene expression with CNVs and methylation from 74 ovarian cancer samples from TCGA (Table 2). There was 1 factor specific to gene expression, 4 to CNVs, and 19 shared factors when the upper bound of 60 factors was set. The largest factor loadings from both CNV and gene expression are clustered around the same region of chromosome 8. Chromosome 8q arm is well associated with disease progression in human cancers. Well-known gene E2F5 (8q21.2), an important cell cycle regulator, is reported in ovarian cancer. Gene expression and methylation joint analysis highlighted SPON1 gene, which is predominantly methylated at its CpG site and is associated with hallmarks of ovarian cancer.^67^

#### Correlation-based approach

##### CNAmet

Louhimo et al, implemented a software package CNAmet for integrative analysis of copy number alteration, DNA methylation, and gene expression data. All data sets should have the same set of samples.^68^

CNAmet consists of 3 major steps: (1) weight calculation which links expression values to copy number and methylation; (2) score calculation step which combines the weights to make 1 score per gene; and (3) significance evaluation which determines the statistical significance of the assigned score with corrected *P* values. The score helps to identify genes that are amplified, hypomethylated, and upregulated or deleted, hypermethylated, and downregulated.^68^

Using the TCGA GBM data of 50 patient samples (Table 2), CNAmet revealed a synergistic effect of DNA methylation and copy number alterations on gene expression for several known oncogenes (such as MDM2, EGFR, and PDGFRA) as well as novel candidate oncogenes. It also showed that patients with hypomethylated EGFR had marginally better prognosis than patients with hypomethylated and amplified EGFR.^68^

#### Other multivariate approaches

##### Multiple co-inertia analysis (MCIA)

The approach used by multiple co-inertia analysis (MCIA) can also help in deriving disease insights. The data sets need not have a common set of features. Analysis of gene expression data (generated on 4 microarray platforms) and protein expression data (generated on liquid chromatography-tandem mass spectrometry [GeLC-MS/MS] platform) for 58 cell lines of NCI-60 panel derived from 9 different tissues (brain, blood and bone marrow, breast, colon, kidney, lung, ovary, prostate, and skin) (Table 2) using MCIA reported that 6 cell line types, central nervous system, leukemia, colon, renal, ovarian, and melanoma, were segregated largely according to their tissue of origin.^53^

Features specific to a cancer cell line were extracted by examining the feature space of genes and proteins that were projected in the same direction and space as the cell lines were. Ingenuity pathway analysis (IPA) on the cell line–specific features revealed significant canonical pathways relevant to the cell lines. For example, the leukocyte extravasation signaling pathway was significantly enriched in leukemia features, whereas melanoma development and pigmentation signaling pathway was enriched in melanoma genes.^53^ In summary, these observations highlight the potential of the MCIA method to derive insights into disease biology.

##### Joint and individual variation explained (JIVE)

Joint and individual variation explained was applied on 234 GBM samples from TCGA (Table 2) containing miRNA and gene expression data.^54^ The joint structure contributes to higher variation in miRNA (23%) than in gene expression (14%), whereas the gene expression data had a considerable amount of individual variation (58%) that is not related to miRNA. However, the joint model showed better classification of samples based on their subtypes than individual structures. POSTN gene, one of the genes with the largest loading weight in joint structure, encodes the protein Periostine which is highly reported in cancerous cell. Downregulation of this gene by miR-219 has been linked to survival and disease progression in GBM.^54^

##### Sparse multi-block partial least squares (sMBPLS)

In this method, a sparse version of PLS is used to decompose the multi-omics data sets into small regulatory blocks called “multi-dimensional regulatory modules” (MDRMs).^69^ Partial least squares is a type of regression method that helps in identifying the relationship between input variables and response variables. Sparse multi-block partial least squares allows multi-block input containing multiple regulatory omics data sets, such as CNV, DNA methylation, and miRNA expression that regulates the gene expression. Gene expression data are used as the response variable. The method aims to identify a subset of genes in a subset of samples from input data sets that jointly explain the expression of genes (response variables) in these samples. These subsets of genes are termed as MDRMs. Sparse multi-block partial least squares aims to identify the driving parameters that optimize the covariance between the input and response data. To apply the sparsity constraint to make negligible coefficients to zero, Lasso penalization is used.^69^

This method was applied on 230 ovarian cancer samples with CNV, DNA methylation, miRNA expression, and gene expression from TCGA (Table 2). The top 100 regulatory modules were identified for further downstream analysis. Forty-eight of the 100 modules were functionally homogeneous, thus indicating the advantage of suing md-modules in clustering relevant features from different regulatory layers. The modules identified the important genes/miRNAs that have been previously reported in ovarian cancers. They also lead to statistically significant interaction networks, thus further validating the functional homogeneity of the identified modules. Furthermore, using IPA, the key regulatory network that affects AKT1 (using genes from module 61) and EGR1 (using genes from module 4) is shown.^69^ This shows the application of sMBPLS in deriving mechanistic details using multi-omics data sets.

##### Thresholding singular value decomposition (T-SVD)

Thresholding singular value decomposition regression (T-SVD) method helps in identifying the regulatory mechanisms between 2 omics data sets, especially when the regulatory features are larger than the measured samples. The regulatory data like miRNA can be used as the predictors, and gene expression data are the response variables through a hidden layer of regulatory programs. The method uses sparsity constraint with the assumption that only a small set of predictors affect the regulatory programs and each program regulates only a small set of response variables. The Bayesian information criterion (BIC), widely used for model selection, is modified to address small set of samples in this method.^70^

This method was used to mine the regulatory mechanisms in ovarian cancer using the miRNA, long noncoding RNA (lncRNA), and gene expression data of 487 samples from TCGA (Table 2). Using miRNA-gene data, regulatory program 1 captured the immunoreactive and proliferative subtypes of ovarian cancer. One of the features, miR-142, reported in other cancers, is shown as the strongest feature. Program 3 with miR-29b and let-7 is also suggested to play important regulatory roles in ovarian cancer.^70^ As per this method and study, the strongest lncRNA was found in the antisense strand of DEPDC1 and another was associated with HMGA2.

##### Joint NMF

This factorization framework identifies correlative modules from multiple data sets (of same samples) to derive md-modules that reveal underlying many layers of regulatory factors.^58^ The method projects the multiple data on a common co-ordinate space wherein the variables highly weighed in the same direction are grouped together into an md-module. To assess whether the vertical correlations within an md-module are significant, Pearson correlation was used between 2 matrices with same row dimensions. The md-modules can facilitate understanding of complex mechanistic details that underlie clinical conditions and can also help in stratification of patients into clinically relevant groups.^58^

The joint NMF method resulted in 200 md-modules using DNA methylation, miRNA expression, and gene expression of 385 ovarian cancer samples from TCGA. Ninety-three percent of the md-modules were functionally homogeneous. Of the 200 modules, 75 showed significant overlap between the genes and their methylation markers within the same module. The modules show significant enrichment of KEGG pathways such as transforming growth factor β signaling, hedgehog signaling, and bladder cancer pathways that are well associated with ovarian cancer. The modules help in deciphering the underlying causal mechanisms in ovarian cancer. For instance, MD module 119 shows synchronous association among the epigenetic regulators, genes, and posttranslational regulation of bladder cancer pathway of KEGG. The clinical associations of the modules helped in stratifying samples based on phenotype-specific modules. For example, 13 patients associated with md-module 166 showed significantly poor survival outcome in which the genes were mostly associated with cell cycle checkpoints and nuclear division.^58^

Platforms such as tranSMART,^71^ Instant Clue,^72^ and MathIomica^73^ provide an open framework to build analysis pipelines using wide array of methods and tools to perform integration and analysis of multi-omics data sets. These platforms allow customization of tools and pipelines as per the requirement and aids in data management and analytics of high-throughput data.

### Other applications of integrating multi-omics data

We have discussed the wide array of application of integrating multi-omics data sets and the tools that help in deriving meaningful results using this approach. The application of integrative analysis is growing, and recent studies have shown more applications that could bring about a revolutionary change in the field of diagnosis, prognosis, and treatment of diseases. Here, we discuss few more strategies of integrative approach that helps in advancement of treatment scenarios.

#### Personalized medicines

Liang et al show a strategy to leverage multi-omics data to identify personalized driver genes. Using mutation, mRNA, and protein expression data of a hypermutated (due to MSH2 inactivation) hepatocellular carcinoma patient, they proposed a strategy to identify the driver genes of the disease in this patient.^74^ Their approach aimed at assessing the impact of tumor-mutated allele on the functional activity of the protein. Their strategy involved 3 criteria that help in identifying the driver genes:

1. Near-saturation of the number of significantly mutated genes;
2. Effect of mutation at mRNA/protein level;
3. Causal implication of the genes in cancer development.

Based on the above criteria, they identified 5 driver genes, HNF1A, IDH1, FAH, GNMT, and SPTBN1, in this patient. They further validated their observations through knockout experiments that the genes identified play a crucial role in tumor cell growth and aggressiveness.^74^ Although this approach can provide advancement in personalized therapy, there are few challenges to understand the synergistic effect of the candidate driver genes.

#### Clinical assessment predictions

Athreya et al proposed a workflow that combines physician’s assessment and omics data to build a predictive model of treatment outcomes for depressive disorders that involve complex phenotypes. Their workflow predicted the therapeutic response by integrating mutation and metabolomics with clinical observations such as patient history, and social and demographic data. They identified different top predictors for men and women, suggesting they respond with different biological mechanisms against antidepressants. Also, the top predictors, mostly metabolites, were previously implicated with mood in behavioral sciences, thus validating their approach. They also showed that the accuracy improved with combining genomics data with metabolomics, clinical, and social data.^75^ This integrated approach can help in providing novel therapeutic interventions for disease with complex phenotypes.

#### Risk prediction and clinical outcome

Mankoo et al implemented a multivariate Cox Lasso (L1-regularized Cox proportional hazards) model that helped in the prediction of time to recurrence and survival along with risk predictions in serous ovarian cancer. By the integration of gene expression, miRNA expression, copy number alteration, and DNA methylation data, the model ranked 156 features to be highly associated with tumor recurrence. The integrated features provided better prediction than individual data sets. Progression-free survival and overall survival from clinical data were used as the outcome measures for the prediction of tumor recurrence. The serous ovarian cancer risk prediction model can aid physicians to predict likely disease progression.^76^

### Comparative analysis and benchmarking of tools

Given such a wide spectrum of tools with various underlying mathematical approaches to integrate and analyze multi-omics data sets, a detailed comparison and benchmarking of methods using the same data sets could prove useful. Tini et al, Rappoport et al, and Chauvel et al have performed detailed comparative analysis and benchmarking of few of the unsupervised clustering methods discussed here.^8,10,45^

Tini et al suggest that multi-omics data integration largely benefits with a feature selection step and that SNF is a robust method among SNF, JIVE, MCIA, MFA, and MCCA (part of package PMA). The conclusions are derived based on method execution on 3 different real data sets with different number of omics and simulated data sets to assess the impact of noise, signal strength, subtypes, feature selection, and training parameters.^10^ On the contrary, Rappoport et al^8^ compared 9 different multi-view clustering methods (LRAcluster, K-means, spectral clustering^77^, SNF, rMKL-LPP, MCCA, multiNMF, iClusterBayes, PINSPlus) using 10 different cancer multi-omics (gene expression, DNA methylation, and miRNA expression) data sets from TCGA. In their analysis, rMKL-LPP, MCCA, and multiNMF^78^ performed better than other methods in terms of clinical subtype enrichment.^8^ Chauvel et al performed a thorough comparative analysis of 6 methods, namely, BCC, MDI, iCluster, moCluster, JIVE, and iNMF. Using simulated data, the methods were assessed for their sensitivity, ability to cluster the samples in the correct manner, and to identify common and specific structures across data sets. Furthermore, using TCGA breast cancer data, the methods were compared for their ability to identify the correct subtype of the samples (Basal, HER2, LuminalA and LuminalB) using SNF, RNA, miRNA, DNA methylation, and RPPA data of 348 samples. The study concludes that iCluster, moCluster, and iNMF perform better clustering, even though iNMF lacks sensitivity. The BCC method showed good ability to identify both common and specific structures between the data sets.^45^

## Portals for Visualization and Interpretation of Multi-omics Data Sets

In addition to the aforementioned list of tools/methods that help in integration of multi-omics data sets to derive meaningful and actionable insights, there are a wide array of portals/platforms that help in exploration, visualization, analysis, and interpretation of multi-omics data. There are a multitude of tools like GENEASE,^79^ CGDV^80^ and SLIDE^81^ that provide ease of visualization and interpretation of large biological data sets. However, these tools help in analysis and visualization of single omic data set at a time. In this section, we provide an account of tools that will make a substantial contribution to visual exploration of interplay of multi-omics data in physiology and diseases. Table 3 summarizes the functionality and features of the portals mentioned in this section.

**Table 3.** List of multi-omics data analysis and visualization portals.

Portal name	Omics data supported	Source repository	Analysis of private data	Availability	Reference
cBioPortal	Mutation, copy number, gene expression, miRNA expression, DNA methylation, protein abundance, and clinical data	TCGA and published studies (http://www.cbioportal.org/)	Yes	http://www.cbioportal.org/	Cerami et al^82^; Gao et al^83^
Firebrowse	Mutation, copy number, gene expression, miRNA expression, DNA methylation, protein abundance, and clinical data	TCGA	No	http://firebrowse.org/	NA
UCSC Xena	Copy number, somatic mutation, DNA methylation, gene and exon expression, protein expression, tissue specific expression data, PARADIGM pathway inference, and phenotype data	TCGA, CCLE, ICGC, GTEX, TARGET, and published studies	Yes	https://xena.ucsc.edu/	Goldman et al^84,85^
LinkedOmics	Clinical data, Copy number, miRNA expression, mutation, DNA methylation, gene expression, protein expression and abundance, phosphoproteome and glyco-proteome data	TCGA and CPTAC	No	http://www.linkedomics.org/	Vasaikar et al^86^
3Omics	Gene expression, protein and metabolite abundance	User data driven	Yes	https://3omics.cmdm.tw/	Kuo et al^87^
NetGestalt	Gene expression, mutation, and copy number data	TCGA, CPTAC, and published studies	Yes	http://www.netgestalt.org/index.html	Shi et al^88^
OASIS	Mutation, copy number, and gene expression data	TCGA, CCLE, GTEx, and published studies	No	http://www.oasis-genomics.org/	Fernandez-Banet et al^89^
Paintomics 3	Gene expression, miRNA expression, metabolite and region-specific ChIP-Seq, and Methyl-Seq data	User data driven	Yes	http://www.paintomics.org/	Hernández-de-Diego et al^90^
MethHC	DNA methylation, gene expression, and miRNA expression	TCGA	No	http://methhc.mbc.nctu.edu.tw/php/index.php	Huang et al^91^

Abbreviations: CCLE, Cancer Cell Line Encyclopedia; CPTAC, Clinical Proteomic Tumor Analysis Consortium; ICGC, International Cancer Genomics Consortium; miRNA, microRNA; GTEx, Genotype-Tissue expression; TCGA, The Cancer Genome Atlas.

### cBioPortal

cBioPortal allows exploration, visualization, and analysis of cancer data containing genomic data, copy number alterations, gene expression, miRNA expression, methylation, and protein abundance data. The portal currently contains data from 233 cancer studies for more than 30 different types of cancer. The portal provides summaries of cancer data, download access to data, network visualization and analysis, and correlation between data sets and patient-centric queries in an intuitive user-friendly manner. The portal aims at integrating multiple data types and thus allows specific query-based results. The ease of use and wide spectrum of tools in this portal have resulted in its popularity among researchers.^82,83^ For instance, Rajendran et al^92^ showed the role of OBSCRN gene in breast cancer tumorigenesis by integrating copy number, mutation, methylation, and gene expression data from cBioPortal.

### Firebrowse

The Broad institute–hosted portal allows analysis, visualization, and download of 38 types of cancer data from TCGA. This portal allows data-specific analysis results and visualization of gene profiles across or specific to cancer type. iCoMut feature shows the broad summary of the top genes across available multiple omics data sets for a cancer cohort in the form of heat maps. The genomic variables can be correlated with clinical variables and other data types (http://firebrowse.org/).

### UCSC Xena

This web-based tool allows visualization and analysis of cancer data sets from TCGA, CCLE, and more than 40 published studies along with allowing analysis of user’s data. This tool allows comparison across studies between different omics data sets and with clinical information. Statistical tools available in this tool allow dynamic quantification and significance of associations. The interactive user interface allows exploration of samples by grouping them based on common clinical features.^84,85^

### LinkedOmics

LinkedOmics allows comparative analysis and exploration of TCGA data from 11 158 samples spanning across 32 different types of cancer. The database also contains proteomics data from CPTAC^15^ for selected samples from TCGA. LinkedOmics contains 3 analysis modules—LinkFinder, LinkCompare, and LinkInterpreter. LinkFinder performs association analysis between and within omics data and clinical attributes. LinkCompare allows comparative analysis within and between data sets (omics platforms, tumor types/subtypes) and thus enables pan-cancer analysis. LinkInterpreter helps in transforming the analysis results from former modules into biological interpretation through pathway and enrichment analysis.^86^ This platform has aided in study-specific correlation analysis between TF/miRNA and their target genes that has helped in deriving useful insights into hepatocellular carcinoma^93^ and cervical cancers.^94^

### 3Omics

This web-based portal supports 3 omics types, namely, transcriptomics, proteomics, and metabolomics. 3Omics analysis requires the use of human transcript, protein, or metabolite IDs and their corresponding variations (eg, concentration or intensity levels) under specific experimental conditions (eg, different times, nucleic magnetic resonance shifts [in parts per million], or mass spectrometry mass-to-charge ratios). Users can perform correlation analysis, co-expression profiling, phenotype mapping, pathway enrichment analysis, and GO enrichment analysis on each data set via a single platform.^87^ Inter-omics analysis and visualization of this platform aided in deriving biomarkers and insights into abnormal Savda syndrome treatment using traditional Uyghur medicines (TUM).^95^

### NetGestalt

NetGestalt is a web application that combines multi-omics data over biological networks. NetGestalt reduces the visualization complexity of large biological networks by placing the nodes in a single horizontal dimension based on hierarchical modular architecture. It uses NetSAM, an R package, to derive the hierarchical organization of networks. It contains hierarchical and modular PPIN based on the protein-protein interactions from HPRD (Human Protein Reference Database). It also allows simultaneous visualization of different types of data within the same framework to facilitate data integration. NetGestalt allows multi-scale representation and navigation of the data, statistical analysis, pathways, and cross-data comparisons in an intuitive manner.^88^ The coexpression module of NetGestalt helped in showing that proteomics outperforms transcriptome in coexpression studies and thus integration of protein profiles could be useful in disease studies.^96^

### OASIS

OASIS is a web-based analytical platform developed by Pfizer for exploration, analysis, and visualization of cancer multi-omics data. This portal contains mutation, copy number, and gene expression data across 55 cancers from TCGA, CCLE, GTEx, and few published studies. Data Summary module provides an overview of all data sets along with exploration of individual data sets. Database Search module provides an interface to build custom queries against all data sets. This BioMart framework^97^ based portal allows pan-cancer exploratory analysis in an easy-to-use fashion to cater to the needs of cancer research community.^89^

### Paintomics 3

This web-based tool offers exploratory tools for visual exploration of multi-omics data sets. It allows processing of gene expression data (NGS and microarray), metabolite data, and region-specific data like ChIP-Seq, Methyl-Seq, and so on. The multi-omic features are mapped to KEGG pathways to create a multi-omics pathway network that allows interpretation of biological significance of the data.^90^ The authors depict the application of their tool in identifying significant pathways and potential mechanisms using transcriptomics, methylation, and histone modification data of human reprogramming of immortalized fibroblasts.^98^

### MethHC

MethHC is a database as well as analysis portal for DNA methylation, gene expression and miRNA expression for 18 human cancers from TCGA. It provides a variety of graphical visualization to perform identification of differentially methylated genes, clustering, and correlation analysis. UCSC genome browser, miRStart, and KEGG pathways are integrated to further enhance the interpretation of results. This portal has helped in deriving important observations like aberrant DNA methylation of miR-31, miR130a, let-7a-3/let-7b, and miR-155 gene promoters has led to silencing of miRNA in breast cancer.^91^

## Challenges in Multi-omics Data Integration and Future Perspectives

Integration of multi-omics data set to derive holistic understanding of biological processes and diseases comes with its share of challenges. The underlying heterogeneity in individual omics data, large size of data sets leading to compute intensive analysis, and lack of studies that help in prioritizing the diverse set of tools make multi-omics data integration and analysis a challenging task. Multi-omics data are generated using wide range of platforms, and hence the data storage and formats vary considerably. Most of the multi-omics integrative analysis tools require data to be in specific formats (mostly in Feature X Sample matrix), and therefore the individual omics data need preprocessing. The preprocessing step includes data filtering, systematic normalization, removal of batch effects, and quality checks. It becomes imperative to carefully use these preprocessing steps as they have a huge influence on the integrative analysis. For instance, data filtering step plays an important role in filtering the noise and reducing the number of features that go into integrative models as most of the integrative methods are compute-intensive and hence it is a prerequisite to reduce the size of the input data sets. However, deciding appropriate criteria for filtering is challenging because of the lack of universal standards. Perez-Riverol et al have developed a workflow that could guide in feature selection from high -dimensional omics data sets.^99^ In this regard, development of new integrative methods/tools must consider efficient handling of large data sets.

The primary key to any integrative analysis is the right choice of method that can address the biological question of interest. There are studies that perform benchmarking of integrative tools,^8,10,45^ but are not comprehensive enough in terms of choice of tools in the context of biological question of interest. More of such comprehensive studies are needed to guide the community in better understanding of the wide array of tools.

Another dimension that could add value to multi-omics data interpretation is the clinical information. Currently, there is no robust method to integrate omics data with the non-omics data such as clinical metadata.^100^ The recent advances in this field are progressing largely with efforts to reduce the challenges. Further developments in integrative analysis of multi-omics data must aim to ease interoperability of multiple data sets and to develop a framework that can help in seamless analysis of multi-omics data.

## Conclusions

Integrative approach using multi-omics data is a powerful strategy to decipher the mechanistic details of the information flow in a cell. Currently, there are a wide array of tools and methods available in the public domain to integrate multi-omics data sets to derive meaningful insights. We have discussed in detail the approach and applications of various integrative methods in this review. We also provide a brief account of multi-omics data repositories, visualization portals, and challenges in integration of data sets. As the tools and methods are largely isolated, there is a need to have a uniform framework that can effectively process and analyze multi-omics data in an end-to-end manner along with easy and biologist-friendly visualization and interpretation.
